# Improving Depot Tracking for Patients in Haringey Community Rehabilitation Team

**DOI:** 10.1192/bjo.2026.11424

**Published:** 2026-06-30

**Authors:** Alicja Beksinska, Tara Amin, Rebecca Long, Rebecca Radmore

**Affiliations:** North London Foundation Trust, London, United Kingdom

## Abstract

**Aims:**

The Haringey Community Rehabilitation team has a high burden of patients on depot antipsychotic medication (n=45). This quality improvement project aims to increase the proportion of depots being administered on time to ensure treatment is delivered as intended, reduce time spent in MDT meetings identifying depot due dates and improve staff-reported ease of tracking depots.

**Methods:**

A baseline audit of depot administration was conducted over four weeks in November 2025. Weekly counts of missed depot administrations found that, on average, 16% of depot injections were either missed or not administered on the scheduled date. In response, a spreadsheet tracking tool, “the depot tracker”, was developed which recorded patient name, depot name and dose, date last administered, date next administration due and named carecoordinator. A traffic-light system was used to indicate depot status: green (due that day), orange (due within seven days), and red (overdue). This allowed prompt identification of which depots are due and which are overdue and facilitated weekly forward planning, including cover during care coordinator leave.

The depot tracker was implemented in December 2025 and incorporated into the weekly MDT meeting agenda with resident doctors updating the tracker weekly. A follow-up audit was conducted post-implementation to reassess rates of timely depot administration. Pre-and post-implementation staff surveys were conducted to assess staff views on ease of tracking when depots are due, including when care coordinators are on leave, and whether tracking depots took up time in MDT meetings. The survey gathered quantitative (5-point Likert scale) and qualitative (free text question) data.

**Results:**

Prior to the depot tracker being introduced a weekly average of 16% of depots were not being administered on time. This reduced to an average of 10% of depots being missed, based on eight weeks of post-implementation data. Regarding improving ease of tracking when depots are due there was a 71.4% improvement noted by MDT members (n=10) and a 66.6% improvement in tracking depots when care coordinators are on leave. In addition, there was a 46.8% improvement in time taken during MDT meetings to look up depot information (n=10).

**Conclusion:**

Following implementation of the depot tracker there has been a reduction in frequency of depots being missed, as well as improvements in staff-perceived ease of depot tracking including improved continuity of depot delivery during care coordinator leave, and increased time efficiency of MDT meetings.

